# Predicting Semen Analysis Parameters from Testicular Ultrasonography Images Using Deep Learning Algorithms: An Innovative Approach to Male Infertility Diagnosis

**DOI:** 10.3390/jcm14020516

**Published:** 2025-01-15

**Authors:** Lutfullah Sagir, Esat Kaba, Merve Huner Yigit, Filiz Tasci, Hakki Uzun

**Affiliations:** 1 Department of Radiology, Faculty of Medicine, Recep Tayyip Erdogan University, 53000 Rize, Turkey; lutfullah.sagir@erdogan.edu.tr (L.S.); esatkaba04@gmail.com (E.K.); filiz.tasci@erdogan.edu.tr (F.T.); 2 Department of Medical Biochemistry, Faculty of Medicine, Recep Tayyip Erdogan University, 53000 Rize, Turkey; merve.huner@erdogan.edu.tr; 3 Department of Urology, Faculty of Medicine, Recep Tayyip Erdogan University, 53000 Rize, Turkey

**Keywords:** semen analysis, deep learning, artificial intelligence, testicular ultrasonography

## Abstract

**Objectives:** Semen analysis is universally regarded as the gold standard for diagnosing male infertility, while ultrasonography plays a vital role as a complementary diagnostic tool. This study aims to assess the effectiveness of artificial intelligence (AI)-driven deep learning algorithms in predicting semen analysis parameters based on testicular ultrasonography images. **Materials and Methods:** This study included male patients aged 18–54 who sought evaluation for infertility at the Urology Outpatient Clinic of our hospital between February 2022 and April 2023. All patients underwent comprehensive assessments, including blood hormone profiling, semen analysis, and scrotal ultrasonography, with each procedure being performed by the same operator. Longitudinal-axis images of both testes were obtained and subsequently segmented. Based on the semen analysis results, the patients were categorized into groups according to sperm concentration, progressive motility, and morphology. Following the initial classification, each semen parameter was further subdivided into “low” and “normal” categories. The testicular images from both the right and left sides of all patients were organized into corresponding folders based on their associated laboratory parameters. Three distinct datasets were created from the segmented images, which were then augmented. The datasets were randomly partitioned into an 80% training set and a 20% test set. Finally, the images were classified using the VGG-16 deep learning architecture. **Results:** The area under the curve (AUC) values for the classification of sperm concentration (oligospermia versus normal), progressive motility (asthenozoospermia versus normal), and morphology (teratozoospermia versus normal) were 0.76, 0.89, and 0.86, respectively. **Conclusions:** In our study, we successfully predicted semen analysis parameters using data derived from testicular ultrasonography images through deep learning algorithms, representing an innovative application of artificial intelligence. Given the limited published research in this area, our study makes a significant contribution to the field and provides a foundation for future validation studies.

## 1. Introduction

Infertility affects approximately 15% of the global population, with its prevalence believed to be increasing over time [1]. Recent studies indicate a gradual decline in sperm count over the past two decades [2]. Male infertility accounts for approximately 50% of all infertility cases and is linked to a range of factors, both congenital and acquired, including genetic influences [3,4].

Diagnostic approaches for male infertility include physical examinations, laboratory assessments, and radiological imaging [5]. Despite significant advancements in these methodologies, the etiology of male infertility remains unexplained in approximately 30–40% of cases [6]. While semen analysis is considered the cornerstone of male infertility evaluation, it offers an incomplete representation of the patient’s overall fertility status [7]. The main limitations of semen analysis include patients’ reluctance to provide samples, anxiety associated with hospital environments, variability in results due to interobserver and intraobserver differences, the time-consuming nature of the procedure, and its inability to consistently assess an individual’s fertility potential [8].

Testicular ultrasonography serves as a valuable adjunct to semen analysis, playing a significant role in identifying the causes of infertility and detecting concomitant conditions, such as testicular tumors [5]. The ultrasonographic parameters commonly associated with fertility status include testicular volume and the presence of varicocele [9]. Research has primarily focused on testicular volume, with several studies demonstrating a correlation between testicular volume and steroidogenic function [10,11]. Although the testicular parenchyma is crucial for both spermatogenesis and steroidogenesis, no standardized radiological parameter has been established to directly assess the parenchyma itself [12].

Additionally, studies have investigated various qualitative parameters of the testis, such as ultrasonographic echogenicity and structural homogeneity/heterogeneity [13]. Research suggests that testicular hypoechogenicity in ultrasonography is associated with impaired spermatogenesis, while testicular heterogeneity has been linked to testicular dysfunction in the context of male infertility [14]. A significant limitation of these studies is the operator dependency involved in characterizing the parenchymal structure of the testis during ultrasonography [5]. In addition, conventional ultrasonography has limitations, including the inability to detect some microstructural changes such as testicular parenchymal abnormalities or mild vascular changes and ultrasonography artifacts [15]. The use of artificial intelligence aims to overcome these problems [16].

Image processing using artificial intelligence represents an innovative engineering approach that extracts quantitative features from digital images, providing insights beyond human visual perception. Artificial intelligence applications in reproductive medicine represent a rapidly developing field in recent years and include many innovative technologies. Current studies in this field generally focus on infertility treatment, embryo evaluation, genetic screening, and patient management. Artificial intelligence is also used in various fields of medicine, but its use in male infertility is limited [17]. By applying quantitative analysis to parameters obtained from testicular ultrasonography, AI can reduce subjective interpretation, thus enabling more accurate and reliable assessments of correlations with testicular function [18,19].

The objective of this study was to enhance the utility of scrotal ultrasonography, improve patient assessment, reduce interobserver variability, and facilitate the prediction of semen analysis outcomes and individual fertility status. This was accomplished by employing deep learning algorithms in artificial intelligence to predict semen analysis parameters based on testicular ultrasonography images.

## 2. Material and Methods

This study was conducted with approval from the institutional ethics committee (approval number: 2023/215; approval date: 20 September 2023).

### 2.1. Patient Selection

The study cohort consisted of male patients aged 18–54 years who presented to the Andrology Outpatient Clinic of our hospital with infertility complaints, despite at least one year of unprotected intercourse. Upon presentation, all patients underwent an initial evaluation by a urologist. The inclusion criteria required the absence of the following conditions: substance abuse, history of anabolic steroid use, testicular asymmetry, undescended testis, inguinal hernia, prior genitourinary trauma, acute infections, or any chronic or congenital diseases.

A total of 289 male patients were evaluated. Of these, 1 patient was diagnosed with a testicular tumor, 20 exhibited microlithiasis, and 19 were identified with azoospermia. These individuals were excluded from the study due to conditions that could potentially compromise the reliability of the results. As a result, the final cohort consisted of 249 patients, with a total of 498 testicular images included for analysis.

The weight and height of each patient were measured, and the body mass index (BMI) was calculated. Additionally, a comprehensive diagnostic assessment was conducted, which included a detailed medical history, semen analysis, biochemical evaluation, and scrotal ultrasonography.

### 2.2. Laboratory Tests

Blood samples were collected from all participants between 8:00 a.m. and 12:00 p.m. following an overnight fast. Serum concentrations of sex steroid hormones, including the follicle-stimulating hormone (FSH), the luteinizing hormone (LH), and testosterone (T), were measured using the Chemiluminescent Microparticle Immunoassay (CMIA) method on the Abbott Architect i2000 autoanalyzer (Abbott Laboratories, Abbott Park, IL, USA).

The data were categorized according to the semen analysis criteria established by the World Health Organization (WHO). Values exceeding these thresholds were classified as normal [19].

### 2.3. Semen Analysis

Semen samples were collected through masturbation into sterile containers after a 2–7 day period of sexual abstinence, under appropriate conditions. Semen analysis was performed by two experienced biologists in the Andrology Unit laboratory. The samples were incubated at 37 °C during liquefaction. Semen volume was determined by estimating its weight, assuming a sperm density of 1 g/mL, using the gravimetric method. Sperm concentration (10^6^/mL) was measured using a Neubauer Improved hemocytometer (Shanghai Qijing Biochemical Instrument Co., Ltd., Shanghai, China), and the total sperm count was then calculated. Progressive motility (WHO grades A + B) was assessed, and sperm morphology was evaluated according to Kruger’s strict criteria.

The data were categorized according to the 2021 World Health Organization (WHO) semen analysis reference values. A sperm concentration below 15 million/mL was classified as oligospermia, a progressive motility below 30% as asthenozoospermia, and a sperm morphology below 4% as teratozoospermia. Values exceeding these thresholds were considered normal [20].

### 2.4. Testicular Ultrasonography

Ultrasonography examinations for all patients in this study were performed by a single radiologist using the Samsung RS85 Prestige Ultrasonography device with the LA2-14A linear probe. The testicular preset, THI mode, and 13.0 MHz parameters were standardized for all patients. To ensure consistency and avoid altering grayscale values, the Tissue Gain Compensation (TGC) was kept constant, and the gain settings were not modified for any patient.

Following semen sample collection, all the patients underwent ultrasonography on the same day. Each patient was examined in the supine position, with the penis positioned in the suprapubic region. Initially, grayscale ultrasonography was used to assess the location, contours, echogenicity, and dimensions of both testes.

**Figure 1:**
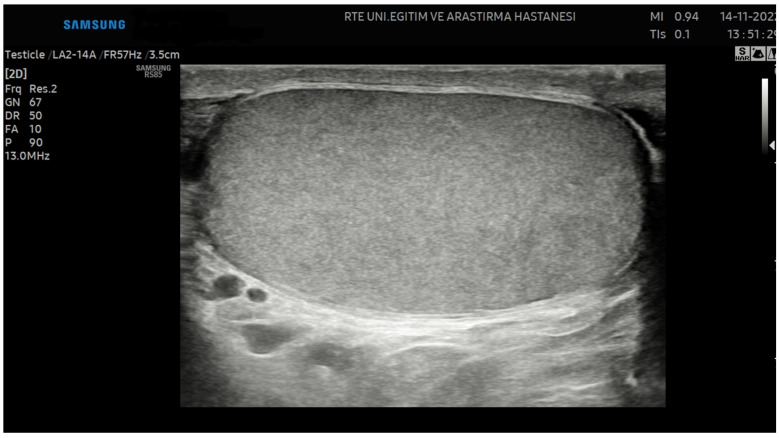
Image of the testis captured along the longitudinal axis.

The short and long axes of the testes were measured, and the testicular volumes were calculated using the following ellipsoid formula: [length (cm) × width (cm) × depth (cm) × 0.71] [9]. However, this ellipsoid formula may not fully account for natural irregularities or asymmetries in testicular shape. Variability in the precision of the measurements and differences in testicular morphology may lead to inaccuracies in volume estimation. In addition, conditions such as testicular atrophy, hydrocele, or the presence of tumors can cause significant deviations from the idealized ellipsoid shape, further limiting the accuracy of the formula [21]. Images were captured along the longitudinal axis of the testis, ensuring that the mediastinum testis was excluded and that the entire testicular contour was encompassed (Figure 1).

### 2.5. Image Preprocessing

**Figure 2:**
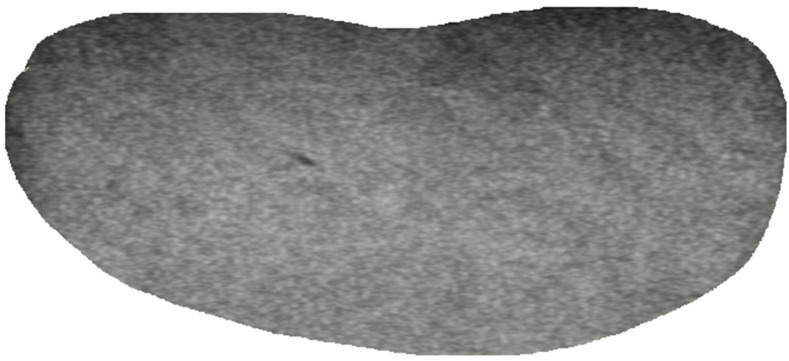
Cropped image of the testis.

The images obtained from the ultrasonography along the longitudinal axis of the testis were converted to PNG format. To remove patient information and minimize the influence of irrelevant areas on the results, the testicular contours were manually outlined and cropped by a single user using a paint program (Figure 2).

### 2.6. Study Design

Based on the data obtained from the semen analysis, the patients were grouped and categorized under the categories of sperm concentration, progressive motility, and morphology. Subfolders were created within each semen parameter folder, dividing them into “low” and “normal” categories. The right and left testicular images of all the patients were then uploaded to the respective folders according to the laboratory parameters.

### 2.7. Deep Learning

After the dataset was compiled for this study, augmentation techniques, including horizontal flipping and 90-degree rotation, were applied to the subgroups with fewer images within each of the three classification categories. The reason why augmentation was applied only to the less represented group instead of the entire dataset was to minimize its impact on the model’s output values. In other words, artificially inflating the model’s performance was avoided. The number of images before and after augmentation is presented in Table 1.

**Figure 3:**
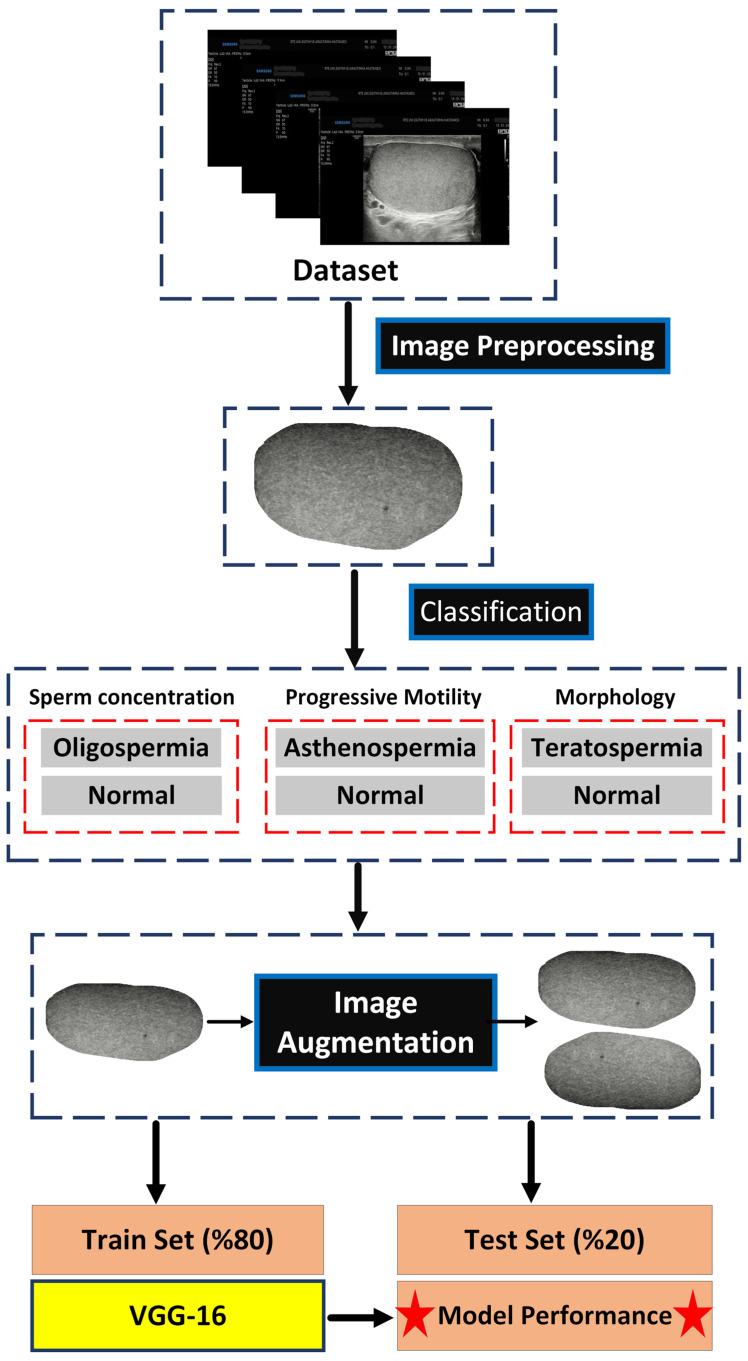
Flowchart of the study.

After image augmentation, the entire dataset was randomly split into 80% training and 20% test sets. The VGG-16 deep learning model, pre-trained on the ImageNet dataset, was used for classification. At this stage, the fully connected and output layers of the VGG-16 model were removed. A new model was created by adding a Global Average Pooling 2D layer and a dense layer with a sigmoid activation function for binary classification. The VGG-16 model was trained using the Adam optimization algorithm. During training, the learning rate and batch size were set to 0.001 and 32, respectively, and the maximum number of epochs was set to 10. The loss function used for the model was binary cross-entropy (binary_crossentropy). The model’s performance was then evaluated using the test set, and metrics such as area under the curve (AUC), accuracy, precision, specificity, sensitivity, and F1 scores were obtained (Figure 3).

## 3. Results

A total of 249 patients were included in this study. Due to missing data in some patients’ hormonal and semen analysis results, each group was matched accordingly (Table 2 and Table 3).

The VGG-16 deep learning architecture was utilized for the classification of sperm parameters, including sperm concentration, progressive motility, and morphology, using ultrasonography images. For each group, 80% of the dataset was used for training, while the remaining 20% was allocated for testing. The number of images used during the training and testing phases is provided in Table 4. The classification results for the test set utilizing the VCG-16 deep learning architecture are presented in Table 5.

**Figure 4:**
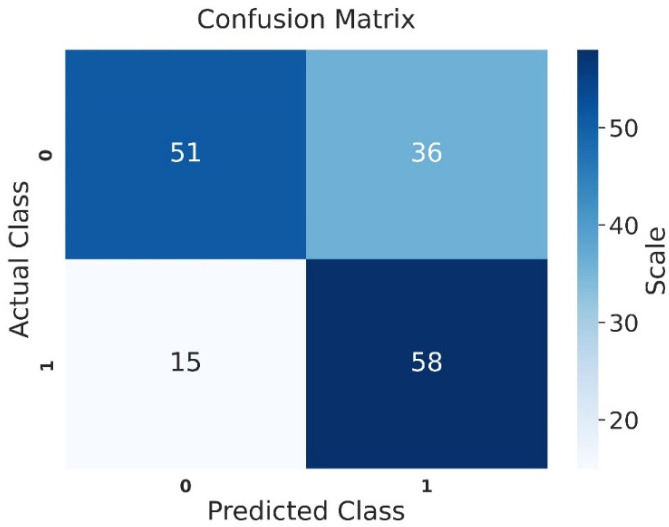
Confusion matrix of the test set for the sperm concentration group (0 = oligospermia, and 1 = normal).

Examining the confusion matrix for the test set of the sperm concentration group, the VGG-16 model correctly classified 51 out of 87 patients with oligospermia. Among 73 patients with normal values, the model identified 58 correctly as normal and misclassified 15 as oligospermic (Figure 4).

**Figure 5:**
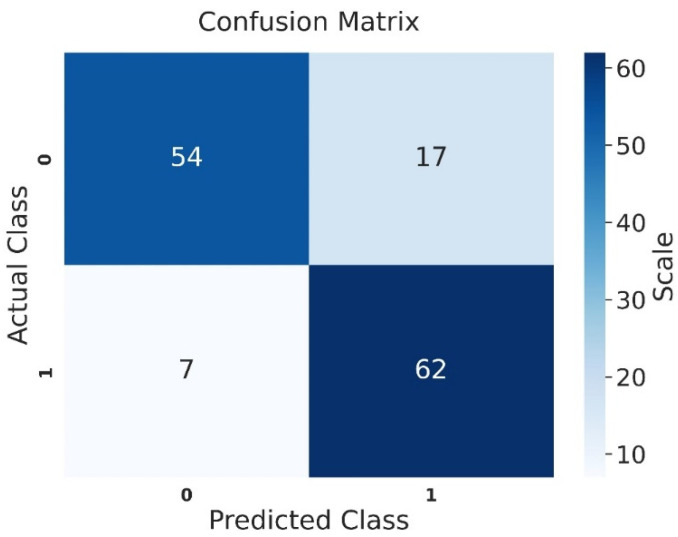
Confusion matrix of the test set for the progressive motility group (0 = asthenozoospermia, and 1 = normal).

Examining the confusion matrix for the test set of the progressive motility group, the VGG-16 model correctly classified 54 out of 71 patients with asthenozoospermia. Among 69 patients with normal values, the model accurately identified 62 as normal and misclassified 7 as asthenozoospermic (Figure 5).

**Figure 6:**
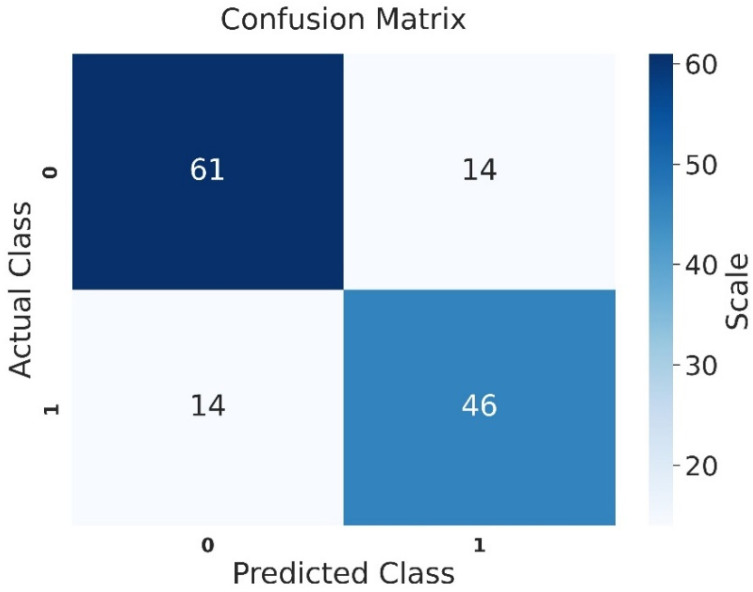
Confusion matrix of the test set for the morphology group (0 = teratozoospermia, and 1 = normal).

Examining the confusion matrix for the test set of the morphology group, the VGG-16 model correctly classified 61 out of 75 patients with teratozoospermia. Among 60 patients with normal values, the model accurately identified 46 as normal and misclassified 14 as teratozoospermic (Figure 6).

**Figure 7:**
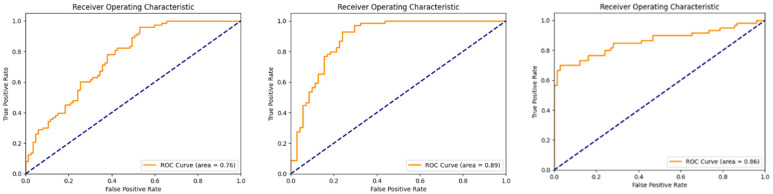
ROC curves for the test set of the sperm concentration, progressive motility, and morphology groups, respectively.

The ROC curves obtained from the test set for the sperm concentration, motility, and morphology groups are presented in Figure 7.

## 4. Discussion

In this study, we explored whether testicular ultrasonography could serve as an alternative to semen analysis by employing artificial intelligence, specifically deep learning techniques. Using data obtained from testicular ultrasound images, we successfully predicted semen analysis parameters. A total of 498 testicular ultrasound images from 249 patients with infertility were collected. The VGG-16 deep learning architecture was utilized to classify semen analysis parameters. The highest area under the curve (AUC) value observed in this study was for the classification of progressive motility, which achieved an AUC of 0.89, effectively distinguishing between the group with asthenozoospermia and that with normal values. This was followed by the morphology classification (teratozoospermia vs. normal), with an AUC of 0.86, and the sperm concentration classification (oligospermia vs. normal), which yielded an AUC of 0.76. The reason why sperm concentration is relatively low compared to the other parameters is that it can be a difficult parameter to directly correlate with ultrasound images. Characteristics such as motility and morphology may be more clearly related to testicular structure and function, whereas concentration may result from more indirect influences.

To the best of our knowledge, this is the first study in the literature to predict semen analysis parameters from ultrasound images using deep learning-based models, contributing both innovation and originality to the existing body of research. Various studies over the years have focused on predicting hormonal levels and semen analysis values based on testicular echogenicity. The general conclusion of these studies suggests that an increase in testicular echogenicity correlates negatively with testicular volume and semen parameters [22,23,24]. However, none of these studies have overcome user subjectivity in ultrasound imaging. This limitation has led to the exploration of alternative approaches. Recently, artificial intelligence has gained considerable attention and offers significant potential in integrating both qualitative and quantitative data derived from ultrasound evaluations. This approach not only mitigates user dependency but also facilitates the acquisition of objective and consistent data [19].

A recent review article reported the findings of a systematic literature search aimed at identifying studies that employed artificial intelligence in testicular imaging. The search revealed a limited number of relevant articles. Among these, the majority focused on the classification of malignant and benign tumors in testicular masses using cross-sectional imaging techniques or predicting lymph node histopathology following chemotherapy. To date, only a single study has explored the application of artificial intelligence to correlate testicular imaging with semen analysis data [25]. In this investigation, De Santi et al. [26] evaluated the relationship between semen parameters and testicular imaging data using radiomics in a cohort of 85 male patients with infertility. The study reported AUC values of 0.62, 0.50, and 0.73 for distinguishing groups based on sperm concentration, motility, and morphology, respectively.

In the study conducted by De Santi et al., the classification performance for sperm concentration, motility, and morphology was limited, likely due to the small sample size and the application of machine learning methods. In contrast, our research utilized a larger patient dataset and employed advanced deep learning algorithms, resulting in significantly higher accuracy rates. While machine learning focuses on analyzing data to identify fundamental relationships, deep learning uncovers more complex data structures, leading to enhanced accuracy [27]. In this study, deep learning demonstrated superior performance compared to machine learning in predicting semen analysis parameters from testicular ultrasound images. By providing user-independent and objective results, this approach offers a more reliable and reproducible method for clinical decision making.

Although high-quality images were obtained using high-frequency probes in this study, a notable limitation was the evaluation of only a single section of the testis. To achieve more comprehensive results, future research could incorporate three-dimensional segmentation of the testicular parenchyma. Another significant limitation was the imbalance in the number of images across subgroups of each semen analysis parameter (low vs. normal). To address this issue, image augmentation techniques were applied to the groups with fewer images, thus balancing the dataset.

Our study was designed as a single-center study, which limits the external validity of the results due to the inability to generalize the findings to broader populations. Patient characteristics, such as demographics, lifestyle factors, or regional health patterns, may vary significantly across different centers, potentially influencing the outcomes. Therefore, to enhance the generalizability of the findings, future studies should involve multi-center collaborations encompassing diverse patient populations.

In our study, specific patient groups, such as those with testicular tumors, microlithiasis, azoospermia, or other conditions which could affect the interpretation of testicular ultrasonography images and semen analysis, were excluded. While this exclusion ensured that the model focused on patients without severe pathologies which could alter the results, it also narrowed the scope of this study. Future research could include these patient groups to evaluate the utility of the model in more complex and heterogeneous populations.

Operator dependency in image acquisition can significantly affect the generalizability of deep learning models, as differences in imaging techniques, positioning, and experience between operators can lead to an inconsistent image quality. This variability can affect the model’s ability to learn the correct features to estimate semen analysis parameters and potentially degrade the model’s performance when applied to images captured by different operators. To minimize this in multi-center studies, standardizing imaging protocols by including images from multiple operators in the training dataset and using data augmentation techniques may help improve the robustness of the model and ensure its applicability in a variety of clinical settings.

## 5. Conclusions

The findings of our study suggest that artificial intelligence has significant potential for predicting specific sperm parameters using only ultrasound imaging in the context of male infertility. This innovative approach could serve as a non-invasive screening tool, offering a potential alternative to traditional semen analysis, which is often criticized for its variability and operational challenges. However, to fully harness the potential of this technology, future research should focus on large-scale, multi-center, and prospective studies to validate its effectiveness and generalizability.

## Figures and Tables

**Table 1:** Number of images before and after data augmentation.

Parameter		Before Augmentation	After Augmentation
Sperm Concentration	Oligospermia	152	456
	Normal	340	340
Motility	Asthenozoospermia	104	312
	Normal	388	388
Morphology	Teratozoospermia	310	310
	Normal	182	364

**Table 2:** Distribution of descriptive, laboratory, and ultrasonography data of the participants.

Parameter	Mean ± SD (Range)
Age	33.7 ± 7.3 (18–54)
BMI	26.9 ± 4.4 (16–43)
FSH (n = 231)	4.6 ± 3.8 (0.3–23.0)
LH (n = 230)	4.3 ± 2.4 (0.8–27.0
Testosterone (n = 231)	428.1 ± 181.8 (47.0–1451.6)
Seminal Volume	3.8 ± 1.7 (0.8–13.0)
Sperm Concentration	40.5 × 10^6^ ± 34.6 × 10^6^ (10^5^–163 × 10^6^)
Progressive Motility	37.6 ± 14.0 (0–68)
Morphology	2.8 ± 2.2 (0–9)
Right Testis Volume	17.4 ± 6.0 (4.0–35.4)
Left Testis Volume	15.9 ± 5.9 (2.2–32.9)

**Table 3:** Correlation between semen parameters and total testicular volume.

	Total Testis Volume	
	Correlation Coefficient (r)	*p*
Volume	−0.067	0.295
Sperm Concentration	0.403	**0.001**
Progressive Motility	0.204	**0.001**
Morphology	0.314	**<0.001**

Spearman correlation test.

**Table 4:** Number of images in the training and test sets after data augmentation.

Parameter	Training	Test
Sperm concentration	638	160
Progressive motility	560	140
Morphology	539	135

**Table 5:** Classification results of the test set using the VGG-16 deep learning architecture.

	AUC	Accuracy	Precision	Specificity	Recall	F1 Score
Sperm concentration	0.76	0.68	0.62	0.59	0.79	0.69
Progressive motility	0.89	0.83	0.78	0.76	0.90	0.84
Morphology	0.86	0.79	0.77	0.81	0.77	0.77

## Data Availability

The original contributions presented in this study are included in the article; further inquiries can be directed to the corresponding author.
